# Oxylipin KODA enhances the early growth of rice (*Oryza sativa* L.) under low-temperature stress at night to simulate a natural temperature condition

**DOI:** 10.5511/plantbiotechnology.24.1218a

**Published:** 2025-03-25

**Authors:** Mineyuki Yokoyama, Takamitsu Kurusu, Hirokazu Ohno, Ohji Ifuku, Rayan Harada, Yuichi Tada

**Affiliations:** 1 Organization for the Strategic Coordination of Research and Intellectual Properties, Meiji University, Higashimita 1-1-1, Tama-ku, Kawasaki, Kanagawa 214-8571, Japan; 2 Department of Mechanical and Electrical Engineering, Suwa University of Science, 5000-1 Toyohira, Chino, Nagano 391-0292, Japan; 3 Research and Development Division, Maruzen Pharmaceuticals Co., Ltd., 5000-1 Toyohira, Chino, Nagano 391-0292, Japan; 4 Avisa Co., Ltd., 1-2-36, Kajino-cho, Koganei, Tokyo 184-0002; 5 School of Bioscience and Biotechnology, Tokyo University of Technology

**Keywords:** KODA, *OsDREB1B*, oxylipin, *PBZ1*, rice

## Abstract

α-Ketol octadecadienoic acid (KODA), an oxylipin, has stimulatory effects on flowering, rooting, and resistance to pathogens. It also increases the yield of rice, *Oryza sativa* L. Here we examined the effects of KODA on the early growth of rice under various temperature conditions. KODA was applied by imbibing seeds in 1 µM KODA solution overnight. KODA treatment did not promote the growth at 25°C or 28°C, which are appropriate temperatures for rice cultivation. At a constant temperature of 15°C, seedling growth was poor, and KODA application did not promote seedling growth. On the other hand, at a night temperature of 15°C and day temperature of 25°C, KODA prominently enhanced the growth. We analyzed the transcript levels of several marker genes associated with chilling signaling and stress tolerance in rice. The expression of the *dehydration-responsive-element-binding protein 1*/*C-repeat binding factor* (*DREB1*/*CBF*), which regulate the expression of many stress-responsive genes was promoted. The expression of the *late embryogenesis abundant* (*LEA*), which has a DRE/CRT *cis*-element, was also increased by KODA treatment. Additionally, the expression of the *b-amylase 4* (*OsBMY4*), which is important for starch degradation during cold-stress adaptation in rice, and that of the *probenazole-induced protein 1* (*PBZ1*), a molecular marker in the rice immune response, were significantly elevated in KODA-treated rice. Thus, the enhanced growth of KODA-treated rice under chilling stress may be attributed, at least in part, to the enhanced transcriptional regulatory network mediated by *DREB1*/*CBF* genes and sugar metabolism, including starch degradation mediated by abscisic acid.

α-Ketol octadecadienoic acid (KODA), an oxylipin, is generated from linolenic acid by 9-specific lipoxygenase, while jasmonic acid (JA) in the same group is synthesized by 13-specific lipoxygenase (Yokoyama 2005). Oxylipins could be produced when plants are exposed to abiotic stress, such as drought, cold and ozone (Ali and Baek 2020) because phospholipase is activated by abiotic stresses to detach linolenic acid (Iqbal et al. 2020). KODA was originally found to be released from *Lemna paucicostata*, which was subjected to stresses such as drought, heat and osmotic pressure (Takimoto et al. 1994; Yokoyama et al. 2000). Both KODA and JA, are stress-related components, but have different activities. Reactants of KODA and norepinephrine showed a strong flower-inducing activity in *L. paucicosta*, strain 151 (Yamaguchi et al. 2001; Yokoyama et al. 2008), but JA did not (Yokoyama et al. 2000). KODA shows various promotive effects on growth in plants under some stress conditions, while JA generally imparts stress tolerance to plants at the cost of growth (Wasternack and Hause 2013). KODA promoted flowering in such species as *Dianthus caryophyllus* (Yokoyama 2005), *Malus domestica* Borkh (Kittikorn et al. 2010, 2011), *Citrus unshiu* Marc (Nakajima et al. 2011), and *Pharbitis nil* (Ono et al. 2013) under weakly inductive conditions for flowering. In studies on the flower-promoting effects of KODA, the essential action of KODA has been elucidated to be involved in the formation of the tissue of both floral and vegetative buds (Nakajima et al. 2011, 2016; Sakamoto et al. 2012). The apparent endodormancy-breaking effects of KODA spray (Sakamoto et al. 2010) may also be involved in a promotive effect on the early growth of floral bud tissue. KODA also induces resistance against plant diseases and pests. KODA induces gene expression for systemic acquired resistance (Endo et al. 2013). KODA applied to grape berry before injection of a pathogen (*Glomerella cingulate*), increased endogenous abscisic acid (ABA), JA and phaseic acid, and then significantly suppressed the development of infection (Wang et al. 2016). KODA was recently reported to induce the defense activity against insect herbivory (Yuan et al. 2023). Such activity to induce resistance against plant diseases and pests would be important for growth in the field. Uno et al. (2021) reported that a single dose of *Lemna* compost® (Maruzen Pharmaceuticals Co., Ltd.) at the procedure of seed imbibition in water before cultivation increased the yield of rice. SPAD value in rice treated with the compost was higher than the control at the maximum-tiller-number stage. *Lemna* compost® contains KODA component at about 0.5 mmol kg^−1^, and the seeds were imbibed in a solution containing 0.7 µM KODA (Uno et al. 2021). We focus on the possibility that KODA treated rice was more resilient under environmental stress. We report here the effects of KODA on the early growth of *Oryza sativa* L. ‘Koshihikari’ under low-temperature stress at night to simulate a natural temperature condition.

KODA was synthesized using *Escherichia coli* transformed with *LpLOX* and *LpAOS* isolated from *L. paucicostata*, strain SH, which produces large amounts of KODA (Takagi et al. 2024). KODA was stocked in ethanol at −80°C until use. We used a plug tray of 2.5 cm for sowing the seeds of rice. The tray was cut out leaving 64 wells (8×8). Each well was packed with KUMIAI-NIPPI-ENGEIBAIDO No.1 (Nihonhiryo Co., Gunma, Japan), but seeds were sown in only 32 wells as described below. The seeds of *O. sativa* L. ‘Koshihikari’ were imbibed overnight in 1 µM KODA solution containing 0.005% ethanol or water containing only 0.005% ethanol (control). After rinsed 3 times by tap water, the seeds were put in the soil packed in a plug tray. KODA-treated and control seeds were sown as a pair, and the pair wells were arranged to provide light and air circulation to all plants evenly. We examined the effects of KODA on rice growth under the condition simulating the environment in May in Kanazawa city, Ishikawa prefecture, where we had previously conducted the yield test of rice with KODA treatment. The average lowest and highest day temperature in mid-May was 14.1°C and 23.1°C, respectively, in Kanazawa city according to the data of Japan Meteorological Agency for 2023 and the daylength was approximately 14 h in mid-May. To simulate this condition, we grew the plants in a 14 h light condition (120–140 µmol/m^2^/s) at 25°C and 10 h dark condition at 15°C (25°C/15°C). In other experiments, we grew plants under the same light/dark condition at a constant temperature of 25°C, 28°C or 15°C. In all cases the plants were cultivated in the NK system Biotron (HCLP 1240 FL-3-5L) (Nippon Medical & Chemical Instruments Co., Ltd., Osaka, Japan). The cultivated plants were harvested after 1 month (at 25°C or 28°C) or about 2 months (25°C/15°C or continuous 15°C). For gene expression analysis, the rice seedlings were sampled at 35th day of cultivation at 25°C/15°C. The plant samples were immediately frozen with liquid nitrogen in microtubes and stored at −80°C. Total RNA was extracted using NucleoSpin RNA Plus kit according to the manufacturer’s instructions (Macherey-Nagel). First-strand complementary DNA (cDNA) was synthesized from 500 ng of total RNA with ReverTra Ace® qPCR RT Master Mix with gDNA Remover (TOYOBO). Real-time PCR was performed using a Bio-Rad CFX Connect Real-Time System (Bio-Rad) with the THUNDERBIRD® SYBR qPCR Mix (TOYOBO) and the specific primers (Supplementary Table S1). Relative mRNA levels were calculated using the 2^−ΔΔC_T_^ method and normalized to correspond to an *OsUbiquitin5* gene (Os01g0328400) level. The relative level of each gene in DMSO control was standardized as 1.

We examined the effects of KODA at various temperatures. KODA did not affect the fresh weight or maximal length of roots and leaves, during a 5-week observation at the constant temperature of 25°C (Figure 1) and 28°C (Supplementary Figure S1). At the constant temperature of 15°C the growth of the seedlings, especially roots, was poor even after 9 weeks. At the constant temperature of 15°C, KODA did not promote the growth of the roots or leaves (Figure 1). However, KODA clearly increased the fresh weight of both roots and leaves of *O. sativa* L. after 9 weeks of cultivation under the day/night temperature of 25°C/15°C (Figure 2). In previous studies, the effects of KODA on rice growth were examined in the field (Uno et al. 2021), where the photon flux density would be much higher than in the indoor artificial light condition. We also examined the effects of KODA at a lower light irradiance of 80–100 µmol/m^2^/s for 6 weeks followed by 120–140 µmol/m^2^/s for 3 weeks. KODA treatment clearly promoted the growth as well as under the condition kept at 120–140 µmol/m^2^/s for 9 weeks (Supplementary Figure S2). Although KODA was applied by imbibing seeds in 1 µM KODA solution overnight in this experiment, 10 µM KODA given for only 1.5 h, also showed the same effect, or even increased the fresh weight of both roots and leaves (Supplementary Figure S3).

**Figure 1:**
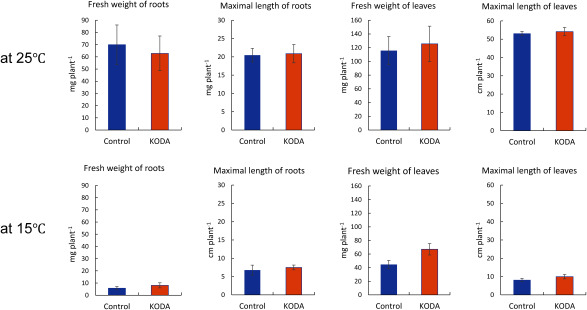
Figure 1. The growth of *O. sativa* L. at the constant temperature 25°C or 15°C. The seeds of *O. sativa* L. were imbibed in 1 µM KODA solution or tap water (control) overnight. Both solutions contained 0.005% ethanol. Seeds were sown in the soil packed in the wells of 2.5 cm plug trays. The buried seeds were arranged as KODA-treated and control pairs. Sixteen pairs were arranged in 64 wells (See text). The plug tray was put on a plastic tray and irrigated from the bottom through stored water, which was supplied as required. They were cultivated at 25°C for 5 weeks or 15°C for 9 weeks under a 14 h-light/10 h-dark condition. Light irradiance was 120–140 µmol/m^2^/s. In the experiment at 25°C, seven of the sixteen pairs were used for data analyses; the seedlings were not used when the germination of one of the pair had been delayed or growth was abnormal. In the experiment at 15°C, all the seedlings growing normally at the 9th week (*n*=7 or 9) were used for analyses because of poor growth and survival of only two pairs. The whole root fresh weight, the longest root length, the whole leaf fresh weight and the longest leaf length were measured after harvesting. Error bars represent SE.

**Figure 2:**
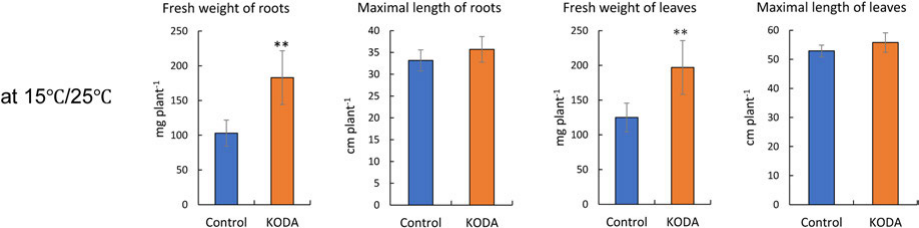
Figure 2. The growth of *O. sativa* L. under 14 h light at 25°C and 10 h dark at 15°C. Plants were harvested after 9-week cultivation and the whole root fresh weight, the longest root length, the whole leaf fresh weight, the longest leaf length was measured. Other cultivating conditions were the same as shown in Figure 1. Error bars represent SE (*n*=10). The asterisk** shows significant difference from the control at *p*<0.01.

The dehydration-responsive element-binding (DREB) proteins/C-repeat binding factors (CBFs) are part of the APETALA2 (AP2) family of transcription factors that bind to the DRE/CRT *cis*-element (Yamaguchi-Shinozaki and Shinozaki 1994) and regulate the expression of various stress-responsive genes (Maruyama et al. 2014). There are over 40 types of stress-inducible genes containing the DRE/CRT *cis*-element (Seki et al. 2001), suggesting that the transcriptional regulatory network mediated by *DREB1*/*CBF* genes is crucial for enhancing the chilling-stress tolerance in plants. To elucidate the mode of action of KODA on transcriptional regulatory networks, we analyzed the transcript levels of several marker genes associated with chilling signaling and stress tolerance in rice after the KODA treatment. The expression level of *OsDREB1B* was markedly elevated in KODA-treated rice compared to the control (Figure 3). A similar pattern was also observed in the expression of the *OsDREB1A* gene. *OsLEA14*, which has the DRE/CRT *cis*-element controlled by *OsDREB1*, was highly expressed with KODA treatment (Figure 3). Additionally, the expression of *OsBMY4*, which encodes β-amylase and is important for starch degradation during cold-stress adaptation in rice (Wang et al. 2023), was also up-regulated in KODA-treated plants (Figure 3). Overexpression of *OsDREB1* results in growth retardation under normal growth conditions in exchange for inducing strong tolerance to low temperature stress (Ito et al. 2006). Further studies are needed to determine the relation between the growth and stress-resistance. Interestingly, the expression level of the probenazole-inducible gene (*PBZ1*), a molecular marker in the rice immune response (Midoh and Iwata 1996), was significantly elevated in KODA-treated rice compared to the control (Figure 3). Previous studies have revealed an increase in ABA in KODA-treated grapes infected by a pathogen (Wang et al. 2016). The *PBZ1* gene was also induced in rice after ABA treatment (Kawahara et al. 2016). ABA accumulation enhances the resistance to chilling-stress and sugar metabolism in numerous plant species (Chen et al. 2020; Danieli et al. 2023; Goswami et al. 2022). In pear, the expression of *PbrBAM3* is induced by cold, dehydration, and ABA. Overexpression of *PbrBAM3* in pear and tobacco promoted starch degradation after chilling stress and subsequently improved cold stress tolerance (Zhao et al. 2019). Thus, the enhanced growth of KODA-treated rice in this low-night temperature condition may be attributed, at least in part, to the enhanced transcriptional regulatory network mediated by *DREB1*/*CBF* genes and sugar metabolism, including starch degradation mediated by ABA.

**Figure 3:**
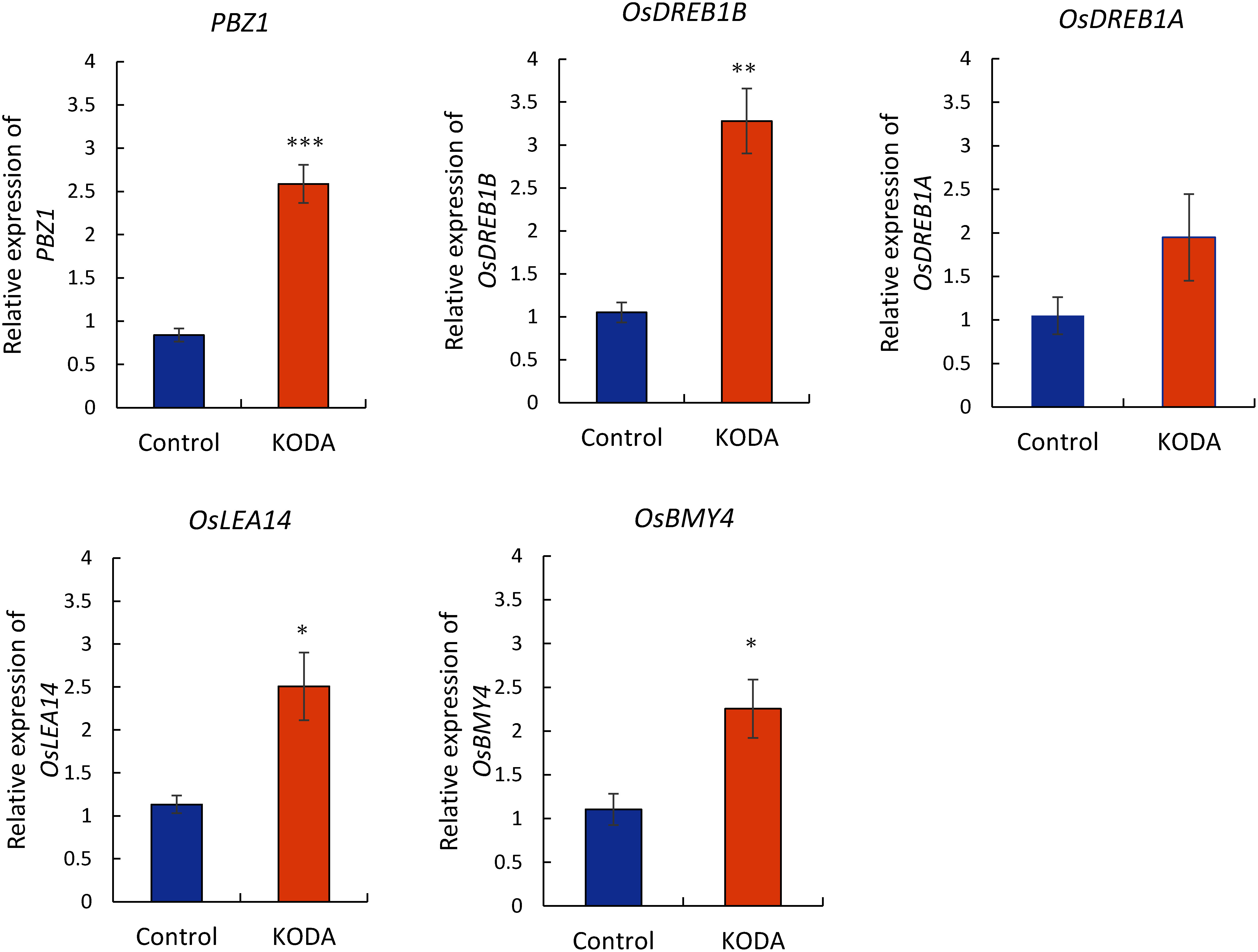
Figure 3. Effects of KODA treatment on gene expression of *O. sativa* L. grown under 14 h light at 25°C and 10 h dark at 15°C. The cultivating conditions were the same as in Figure 2. Plants were harvested after 35 days. Quantitative expression levels of *PBZ1*, *OsDREB1B*, *OsDREB1A*, *OsLEA14*, and *OsBMY4* in KODA-treated rice plants. The expression values of the individual genes were normalized using *UBQ5* as an internal standard. The relative level of each gene in the DMSO control was standardized as 1. Error bars represent SE (*n*=5). *** *p*<0.005, ** *p*<0.01 and * *p*<0.05; significantly different from the control.

## Abbreviations

KODA: 9-hydroxy-10-oxo-12(Z),15(Z)-octadecadienoic acid

## Description of Supplementary Files

Supplementary Table S1. Primers used in this qPCR experiment.

Supplementary Figure S1. Effects of KODA on the growth of *O. sativa* L. at 28°C constant.

Supplementary Figure S2. Effects of KODA on root and leaf fresh weight, the maximum root length and the number of leaf of *O. sativa* L. under low-temperature stress at night for a simulated natural temperature condition (15°C for night/25°C for day).

Supplementary Figure S3. KODA effects were examined when 10 µM KODA was applied for only 1.5 h.
