# Supplementary material for: Oxylipin KODA enhances the early growth of rice (Oryza sativa L.) under low-temperature stress at night to simulate a natural temperature condition
Source: Plant Biotechnol (Tokyo). 2025 Mar 25;42(1):73–7. doi: 10.5511/plantbiotechnology.24.1218a (PMC12622901; doi:10.5511/plantbiotechnology.24.1218a)
Supplement: Supplementary Data [file plantbiotechnology-42-1-24.1218a-s001.pdf]

Supplementary Table S1. Primers used in this qPCR experiment.

Gene name	Accession No.	Primer sequence
OsDREB1A	Os09g0522200	F: ACCTGTACTACGCGAGCTTG<br>R: TAGTAGCTCCAGAGTGGGAC
OsDREB1B	Os09g0522000	F: GATGGCGACGAAGAAGAAGA<br>R: GAACCTGAACCCGTCGTC
OsLEA14	Os01g0705200	F: TGTGACTCGATCCAGCGTAG<br>R: GTTCCTGCTGAGAAGCCATC
OsBMY4	Os03g0141200	F: ACGTGCGTGGAGATGCGCAA<br>R: ACCTGCTCGCGGCACACGTC
PBZ1	Os12g0555500	F: GCGTTTGAGTCCGTGAGAGT<br>R: TCACCCATTGATGAAGCAAA
UBQ5	Os01g0328400	F: ACCACTTCGACCGCCACTACT<br>R: ACGCCTAAGCCTGCTGGTT

Supplementary Figure S1

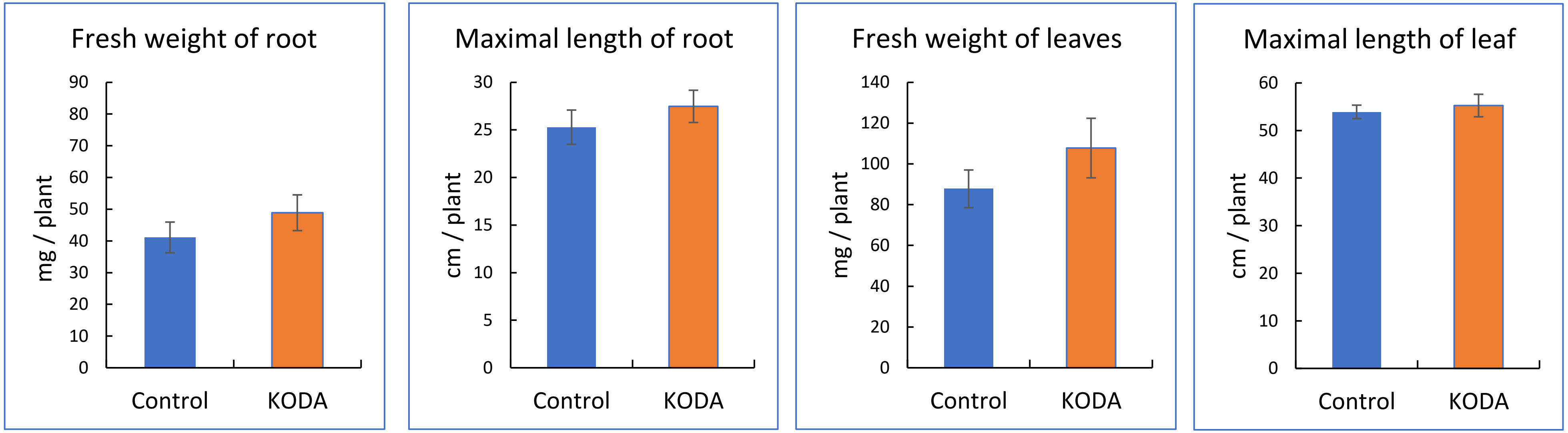

Effects of KODA on the growth of *Oryza sativa* L. at 28ºC constant. KODA (1 µM) was applied by imbibing seeds over night. The rice seedlings were cultivated for one month at 28ºC on light (14 hr)/dark (10 hr) condition. Light irradiance was 80-100 µmol/m^2/s.

KODA failed to promote the growth of the seedlings.

Supplementary Figure S2

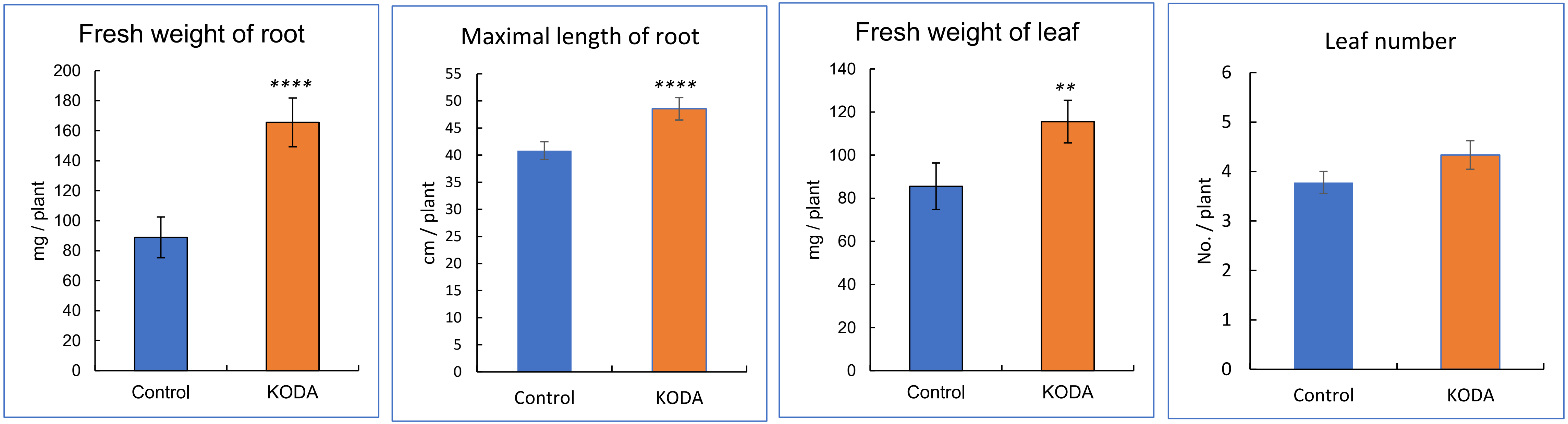

Effects of KODA on Root and leaf fresh weight, the maximum root length and the number of leaf of *Oryza sativa* L under low-temperature stress at night to simulate natural temperature condition (15ºC for night/25ºC for day). In this experiment, lighting condition was 80-100 μmol/m^2/s for 6 weeks before 120-140 μmol/m^2/s for 3 weeks. Other conditions were the same as in Fig. 1. The plants were harvested after 9 weeks.

Root and leaf fresh weight was prominently increased as shown in Fig. 3. The maximum root length was also increased in this experiment. The maximum leaf length was not measured. The asterisk** at *P*<0.01 and **** at *P*<0.001. N=9

Supplementary Figure S3

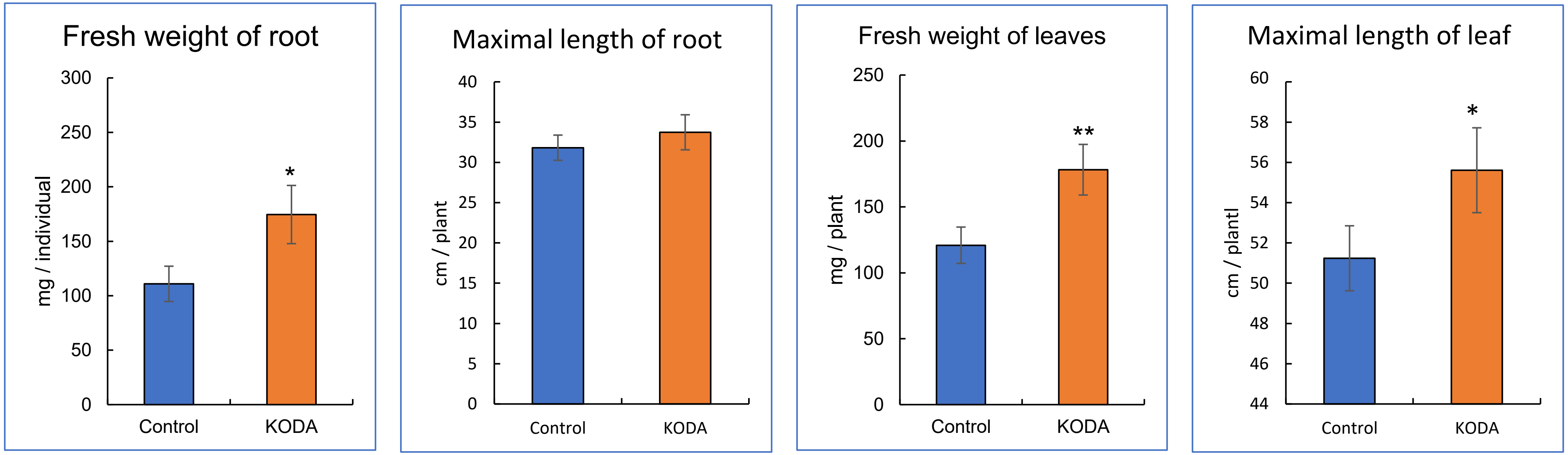

KODA effects were examined when 10 μM KODA was applied for only 1.5 hr. Fresh weight and the maximal length of both root and leaf of *Oryza sativa* were measured after cultivating for 8 weeks in a simulated natural temperature (15°C for night/25°C for day). Light irradiance was 120-140 μmol/m^2/s.

KODA promoted the growth of the seedlings; fresh weight of root and leaf was increased. Maximal length of leaf also increased longer than control.
